# Antioxidant properties of xanthones from *Calophyllum brasiliense:* prevention of oxidative damage induced by FeSO_4_

**DOI:** 10.1186/1472-6882-13-262

**Published:** 2013-10-11

**Authors:** Tonali Blanco-Ayala, Rafael Lugo-Huitrón, Elizabeth M Serrano-López, Ricardo Reyes-Chilpa, Edgar Rangel-López, Benjamín Pineda, Omar Noel Medina-Campos, Laura Sánchez-Chapul, Enrique Pinzón, Trejo-Solis Cristina, Daniela Silva-Adaya, José Pedraza-Chaverrí, Camilo Ríos, Verónica Pérez de la Cruz, Mónica Torres-Ramos

**Affiliations:** 1 Departamento de Neuroquímica, Instituto Nacional de Neurología y Neurocirugía Manuel Velasco Suárez, Insurgentes Sur 3877, S.S.A., México, DF 14269, México; 2 Instituto de Química, Universidad Nacional Autónoma de México, México, DF 04510, México; 3 Laboratorio de Aminoácidos Excitadores, Instituto Nacional de Neurología y Neurocirugía Manuel Velasco Suárez, S.S.A., México, DF 14269, México; 4 Laboratorio de Neuroinmunología, Instituto Nacional de Neurología y Neurocirugía Manuel Velasco Suárez, S.S.A., México, DF 14269, México; 5 Departamento de Biología, Facultad de Química, Universidad Nacional Autónoma de México, México, DF 04510, México; 6 Laboratorio de Bioquímica Muscular, Instituto Nacional de Rehabilitación, S.S.A., México, DF 14389, México; 7 Unidad del Bioterio, Facultad de Medicina, Universidad Nacional Autónoma de México, México, DF 04510, México; 8 Unidad Periférica de Neurociencias Facultad de Medicina UNAM-INNN, México, DF 14269, México

**Keywords:** Xanthones, Antioxidant capacity, ROS production

## Abstract

**Background:**

Reactive oxygen species (ROS) are important mediators in a number of degenerative diseases. Oxidative stress refers to the imbalance between the production of ROS and the ability to scavenge these species through endogenous antioxidant systems. Since antioxidants can inhibit oxidative processes, it becomes relevant to describe natural compounds with antioxidant properties which may be designed as therapies to decrease oxidative damage and stimulate endogenous cytoprotective systems. The present study tested the protective effect of two xanthones isolated from the heartwood of *Calophyllum brasilienses* against FeSO_4_-induced toxicity.

**Methods:**

Through combinatory chemistry assays, we evaluated the superoxide (O_2_^●—^), hydroxyl radical (OH^●^), hydrogen peroxide (H_2_O_2_) and peroxynitrite (ONOO^—^) scavenging capacity of jacareubin (xanthone III) and 2-(3,3-dimethylallyl)-1,3,5,6-tetrahydroxyxanthone (xanthone V). The effect of these xanthones on murine DNA and bovine serum albumin degradation induced by an OH• generator system was also evaluated. Additionally, we investigated the effect of these xanthones on ROS production, lipid peroxidation and glutathione reductase (GR) activity in FeSO_4_-exposed brain, liver and lung rat homogenates.

**Results:**

Xanthone V exhibited a better scavenging capacity for O_2_^●—^, ONOO^-^ and OH^●^ than xanthone III, although both xanthones were unable to trap H_2_O_2_. Additionally, xanthones III and V prevented the albumin and DNA degradation induced by the OH^●^ generator system. Lipid peroxidation and ROS production evoked by FeSO_4_ were decreased by both xanthones in all tissues tested. Xanthones III and V also prevented the GR activity depletion induced by pro-oxidant activity only in the brain.

**Conclusions:**

Altogether, the collected evidence suggests that xanthones can play a role as potential agents to attenuate the oxidative damage produced by different pro-oxidants.

## Background

**Figure 1:**
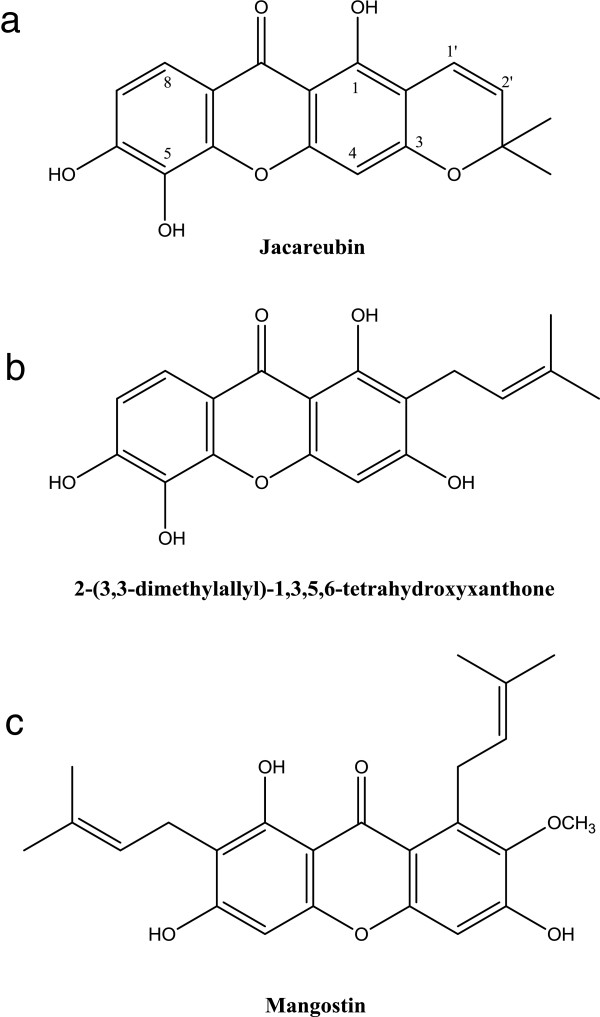
Chemical structures of xanthones: a) jacareubin (III), b) 2-(3,3-dimethylallyl)-1,3,5,6-tetrahydroxyxanthone (V), and c) α-mangostin.

Redox homeostasis is maintained in organisms as an intracellular equilibrium between oxidant and antioxidant levels. An imbalance in favor of pro-oxidants results in oxidative stress which leads cells to damage through the alteration of endogenous macromolecules such as proteins, lipids and DNA [1,2]. Cells possess several antioxidant defense mechanisms designed to maintain homeostasis in response to oxidative stressors; however, under pathological conditions, such antioxidant defenses are depleted, thus eliciting oxidative damage. The most promising strategy to prevent the oxidative damage caused by reactive oxygen and nitrogen species (ROS/RNS) is the use of antioxidant molecules. These compounds can act either as direct antioxidant through free radical scavenging mechanisms or as indirect antioxidants by enhancing the antioxidant status toward both enzymatic and non-enzymatic systems. Different natural compounds have been described as antioxidants, as they are capable to decrease ROS levels in cells and are consequently useful to attenuate aging-related complications [3,4] and for treatment of some human diseases, including atherosclerosis, cardiovascular diseases [5], inflammatory injury [6], cancer [7] and neurodegenerative diseases [8]. Natural antioxidants can therefore serve as innovative tools for preventive medicine. *Calophyllum brasiliense Cambess* is a big tree found in the tropical rain forests from Brazil to Mexico. It has been widely used in Latin American folk medicine to treat a variety of maladies including pain [9], inflammation, diabetes, hypertension [10,11], diarrhea, herpes and rheumatism [12]. Despite its extensive use, only a few biological activities have been reported in the scientific literature. Plants of this species are a rich source of xanthones and coumarins. Particularly, xanthones (9*H-*xanthen-9-ones) are heterocyclic compounds with the dibenzo-γ-pyrone framework (Figure 1) [13]. Several xanthones have been isolated from *Calophyllum brasiliense* heartwood, including jacareubin (III) and 2-(3,3-dimethylallyl)-1,3,5,6-tetrahydroxyxanthone (V). Xanthones from *Callophyllum brasiliense Cambess* have shown to be inhibitors of sulfotransferases, SULT1A1 and SULT2A1 [14], antibiotics [15], and inhibitors of gastric H^+^, K^+^−ATPase activity [16]. Furthermore, it has been reported that xanthones from other sources, like α-mangostin, are effective antioxidants and could be important free radical scavengers [17-21]. This study was conducted to explore the scavenging effects and antioxidant properties of xanthones III and V (Figure 1b and 1c, respectively) isolated from *Callophylum brasiliensis, a species* collected in the Mexican rain forest. Here, we evaluated the activities of these xanthones as scavengers of different ROS in synthetic systems. Also, we tested their effects against oxidative damage induced by FeSO_4_ in brain, liver and lung rat homogenates.

## Methods

### Reagents

*Calophyllum brasiliense Cambess (Clusiaceae)* was collected by J.I. Calzada from the Selva Lancandona, Chiapas, México [22]. Identification was done by the collector, a voucher (JIC-3116), and wood simple (00011-XALw) are deposited in the Xal Herbarium, and Dr. Faustino Miranda Xylarium, both of the Instituto de Ecología, A.C., at Xalapa, Ver. México. Five xanthones (I-V) were isolated from *Calophyllum brasiliense* heartwood; xanthones III and V were obtained from a methanolic extract and were the most abundant constituents, as previously described, and identified by proton nuclear magnetic resonance and mass spectrometry [23]. α-Mangostin was isolated from the dried pericarp of *Garcinia mangostana*, as previously described by Marquez-Valadez et al. [24].

2′,7′-dichlorofluorescein (DCF), 2′,7′-dichlorodihydrofluorescein diacetate (DCFH-DA), FeSO_4_, ascorbic acid, histidine, xylenol orange, ammonium iron (II) sulfate hexahydrate, 2,2′-azinobis-(3-ethylbenzothiazoline-6-sulfonic acid) (ABTS), 2,2-diphenyl-1-picrylhydrazyl (DPPH), bovine serum albumin (BSA), dimethylsulfoxide (DMSO), DL-penicillamine, glutathione (GSH), nitroblue tetrazolium (NBT), phenazine methosulfate (PMS), ethylenediaminetetraacetic acid (EDTA), nicotinamide adenine dinucleotide (NADH), N-acetyl cysteine (NAC), N-2-hydroxyethyl-piperazine-N'-2-ethane sulphonic acid (HEPES), diethylenetriaminepentaacetic acid (DTPA), nicotinamide adenine dinucleotide reduced form (NADPH), Coomassie brilliant blue R, mercaptoethanol, bromophenol blue, sodium dodecyl sulfate (SDS), sodium hypochlorite (NaOCl), hydrogen peroxide (H_2_O_2_) and N,N-dimethyl-4-nitrosoaniline (DMNA) were all obtained from Sigma Aldrich Chemical Company (St. Louis, MO, USA). All other reagents were analytical grade and obtained from known commercial suppliers. Solutions were prepared using deionized water obtained from a Milli-RQ (Millipore) purifier system.

### Animals

Tissue samples were obtained from the whole brain of male Wistar rats (280–320 g) from the vivarium of Facultad de Medicina (UNAM, Mexico City). A total of 40 rats were employed throughout the study. Before they were subjected to experimental treatments, animals were housed five per cage in acrylic cages and provided with standard commercial rat diet (Laboratory rodent diet 5001, PMI Feeds Inc., Richmond, IN, USA) and water *ad libitum*. Housing room was maintained under constant conditions of temperature (25 ± 3°C), humidity (50 ± 10%) and lighting (12 h light/dark cycles). Tissues were collected by decapitation and immediately dissected out on ice and preserved for limited times at −70°C. All procedures with animals were carried out according to the *National Institutes of Health Guide for the Care and Use of Laboratory Animals* and the local guidelines on the ethical use of animals from the Health Ministry of Mexico. During the dissections, all efforts were made to minimize animal suffering. The study was approved by Instituto Nacional de Neurología y Neurocirugía’s ethics committee (30/12).

### Superoxide anion scavenging assessment

Superoxide (O_2_^●—^) scavenging was assessed according to previously reported methods [25] based on the reduction of NBT. The non-enzymatic PMS/NADH system generates superoxide radicals that reduce NBT into a purple-colored formazan. The reaction mixture contained HEPES buffer (20 mM, pH 7.4), 196 μM NADH, 39.2 μM NBT, 3.92 μM PMS and increasing concentrations of Xanthone III or V (0 – 1,000 μM). Final mixture volume was 1.3 mL. After incubation for 5 min at room temperature, the absorbance was taken at 560 nm against an appropriate blank solution. All tests were performed six times in an independent manner. Results are shown as percent of O_2_^●—^ scavenging capacity.

### Hydroxyl radical (OH^●^) scavenging assay

The ability of xanthones III and V to scavenge OH^●^ was estimated through the Fe^3+^-EDTA-H_2_O_2_-deoxyribose system [26,27]. The system contained different concentrations of xanthones III or V (0 – 50 μM or an equivalent volume of vehicle (ethanol, 0.025% final concentration used in the assay)) or distilled water for the control), 0.2 mM ascorbic acid, 0.2 mM FeCl_3_, 0.208 mM EDTA, 1 mM H_2_O_2_, 0.56 mM deoxyribose, and 20 mM phosphate buffer (pH 7.4). OH^●^ radicals were generated by incubating the mixture (final volume 1 mL) at 37°C for 60 min. The iron salt (FeCl_3_) was mixed with EDTA before its addition to the reaction mixture. The extent of deoxyribose degradation by the formed OH^●^ was measured directly in the aqueous phase by the thiobarbituric acid (TBA) test. Briefly, 100 μL of the 0.05 M TBA solution was added to the samples and final solutions were re-incubated in a boiling water bath (94°C) for 10 min. The optical density was estimated at a wavelength of 532 nm in a Genesys 8 spectrophotometer.

### Peroxynitrite (ONOO^—^) scavenging activity assessment

ONOO^—^ was synthesized as previously described [28]. Briefly, 5 mL of an acidic solution (0.6 M HCl) of H_2_O_2_ (0.7 M) was mixed with 5 mL of 0.6 M KNO_2_ on an ice bath for 1 second, and the reaction mixture was quenched with 5 mL of ice-cold 1.2 M NaOH. Residual H_2_O_2_ was removed using granular MnO_2_ prewashed with 1.2 M NaOH, and this mixture was then left overnight at −20°C. The resulting yellow liquid layer on the top of the frozen mixture was collected for the experiment. Concentrations of ONOO^—^ were determined before each experiment at 302 nm using a molar extinction coefficient of 1,670 M^-1^ cm^-1^. ONOO^—^ scavenging activity was measured by monitoring the oxidation of DCFH-DA to DCF by the modified method of Beckman and Crow [28,29]. The reaction mixture (in a final volume of 1.45 mL in 0.1 M phosphate buffer pH 7.4) consisted of 14 μM DTPA plus 36.2 μM DCFH-DA plus the samples exposed to different xanthones III and V at increasing concentrations (0–1,000 μM) and 35 μM ONOO^—^. The optical density was determined at 500 nm in a Genesys 8 spectrophotometer. A probe containing the reaction mixture, but not a sample, was considered as 0% scavenging capacity or 100% of DCFH-DA oxidation by the ONOO^—^ added to the assay. To calculate the ONOO^—^ scavenging ability, the readings of the tubes containing the xanthones or reference compounds were expressed as percentages of DCFH-DA oxidation and converted to percentages of scavenging ability, using as reference the tube with 100% DCFH-DA oxidation [26].

### H_2_O_2_ assay

The ability of xanthones III or V to scavenge H_2_O_2_ was measured by the method described by Long et al. [30]. Briefly, 9 volumes of 4.4 mM butylated hydroxytoluene in HPLC-grade methanol were mixed with 1 volume of 1 mM xylenol orange and 2.56 mM ammonium ferrous sulfate in 0.25 M H_2_SO_4_ to give the "working" FOX reagent. A solution of 75 μM H_2_O_2_ was mixed with different concentrations of xanthones III and V (0–1000 μM) and added with 0.01 mL of HPLC-grade methanol, immediately followed by the addition of 0.9 mL of FOX reagent, vortexed for 5 seconds, and then incubated at room temperature for 30 min (final volume 1.3 mL). Tubes were centrifuged at 15,000 × g for 10 min and absorbance was read at 560 nm against a methanol blank.

### OH^●^-mediated protein degradation

Experiments for detection of OH^●^-mediated BSA oxidation were carried out using a metal-catalyzed reaction based on the method described by Kocha et al. [31] with modifications. A solution of ascorbic acid (1.6 mM)/EDTA (0.8 mM)/(NH_4_)_2_Fe(SO_4_)_2_ (0.8 mM) was prepared in 50 mM phosphate buffer (pH 7.4). Briefly, 1% BSA and 250 μL of the ascorbic acid/EDTA/(NH_4_)_2_Fe(SO_4_)_2_•6H_2_O solution were mixed in the presence or absence of xanthones III and V or α-mangostin (0.5, 1 and 2.5 μM). The generation of OH^●^ was initiated through the addition of 15 μL of 2% H_2_O_2_. In control tubes (without the OH^●^ generator system), H_2_O_2_ was replaced by water. The final volume for all probes was 250 μL. The maximum concentration of EtOH (vehicle) in reaction tube was 0.01%. After 1 h of incubation at room temperature, 250 *μ*L of 20% trichloroacetic acid was added, and the mixture was then centrifuged at 2,236 x *g* for 30 min at 4°C. The supernatants were discarded and the pellets were resuspended in 500 *μ*L of 0.1 M NaOH. To evaluate the oxidative damage to proteins induced by OH^●^, the samples were subjected to SDS-polyacrilamide gel electrophoresis. BSA (50 *μ*g; from resuspended pellets) was mixed (1:1) with loading buffer (10% glycerol, 2% SDS, 25 mM Tris–HCl (pH 6.8), 5% mercaptoethanol, 0.1% bromophenol blue) and heated at 100°C for 1 min. The protein sample (40 μg per well) was loaded in a 12% polyacrylamide gel and the electrophoresis was run at 150 V for 1 h. After running out, gels were stained with 0.2% Coomassie brilliant blue R for 1 h. Images were visualized and captured in a BioRad Gel Documentation System (Gel Doc 1000 BioRad). Protein levels were densitometrically evaluated using the Quantity One Program 4.2 [32].

### OH^●^-mediated DNA degradation

DNA samples were obtained from mice tails and incubated overnight at 55°C in lysis buffer (Tris 100 mM, pH 8.5, 5 mM EDTA, 0.2% SDS, 200 mM NaCl and 100 μg/ml proteinase K). The mixtures were deproteinized with phenol:chlorophorm:isoamilic ethanol (25:24:1). The DNA in the recovered supernatant was precipitated by adding cold isopropanol and washed with 70% ethanol solution, then dissolved in endonucleases-free water for the assay. Experiments for detection of OH^●^-mediated DNA oxidation were carried out by using a metal-catalyzed reaction based on Kocha et al. [31] with modifications. A solution of ascorbic acid (1.6 mM)/EDTA (0.8 mM)/(NH_4_)_2_Fe(SO_4_)_2_ (0.8 mM) was prepared in 20 mM phosphate buffer (pH 7.4) plus DNA, xanthones III or xanthone V, and α-mangostin (0.5, 1 and 2.5 μM). Briefly, 20 μg of purified DNA was mixed with 25 μL of the ascorbic acid/EDTA/(NH_4_)2Fe(SO_4_)_2•_6H_2_O solution, in presence or absence of xanthones and α-mangostin. The generation of OH^●^ was initiated through the addition of 15 μl of 2% H_2_O_2_. In control tubes (without the OH^●^ generator system), H_2_O_2_ was replaced by water. The final volume for all probes was 250 μL. The maximum concentration of EtOH (vehicle) in reaction tube was 0.01%. After 15 min of incubation at room temperature, 50 μL of loading buffer was added to stop the reaction. To evaluate the oxidative damage to DNA induced by OH^●^, 10 μL of the samples were subjected to agarose (2%) gel electrophoresis for 20 min at 90 V. After running out, gels were stained with ethidium bromide (0.1 mg/ml). Images were visualized and captured in a BioRad Gel Documentation System (Gel Doc 1000 BioRad). In order to estimate DNA degradation; the main DNA band per well was densitometrically evaluated using the Quantity One Program 4.2 [32].

### Assay of lipid peroxidation

Lipid peroxidation (LP) was assessed in homogenates of different tissues (brain, liver and kidney) by estimation of TBA-reactive substances (TBA-RS), according to a previous report [33]. Each tissue was homogenized (brain 1:10, liver 1:300, kidney 1:100 w/v) in Krebs buffer (pH 7.4) containing 19 mM NaCl, 5 mM KCl, 2 mM CaCl_2_, 1.2 mM MgSO_4_, 5 mM glucose, 13 mM NaH_2_PO_4_ and 3 mM Na_2_HPO_4._ Aliquots of 250 μL of the homogenate were incubated in the presence of FeSO_4_ (5 μM) and/or xanthone III and V (0.5, 1 and 2.5 μM). The final volume for all probes was 500 μL. All incubations were done at 37°C for 2 h in a shaking water bath. At the end of the incubation time, 500 μL of the TBA reagent (containing 0.75 g of TBA + 15 g of trichloroacetic acid + 2.54 mL of HCl) were added and final solutions were re-incubated in a boiling water bath (94°C) for additional 20 min. Samples were then kept on ice for 5 min and centrifuged at 3,000 x g for 15 min. The optical density of supernatants was estimated at a wavelength of 532 nm in a Genesys 8 spectrophotometer. Concentrations of malondialdehyde (MDA) were calculated by interpolation in a standard curve of MDA, constructed in parallel. Results were expressed as percent of LP vs. control.

### Determination of ROS in homogenates

ROS were detected by DCF fluorescence [34,35]. Aliquots (500 μL) of the homogenates (brain, liver and kidney) were incubated in the absence (control) or presence of 5 μM FeSO_4_ and/or xanthones III or V (0.5, 1 and 2.5 μM) at 37°C in a shaking-water bath for 2 h. The final volume for all probes was 1 mL. Then 100 μL of 75 μM DCF were added to samples and incubated for 30 min in the abscence of light. Finally, samples were centrifuged at 6,000 x g for 15 min. ROS were conventionally detected in supernatants by fluorescent spectrometry in a Perkin-Elmer LS50 spectrometer at 488 nm (excitation wavelength) and 532 nm (emission wavelength). Results were expressed as percent of ROS formation vs. control.

### Glutathione reductase (GR) activity

GR activity in forebrain, liver and kidney homogenates was assayed using oxidized glutathione (GSSG) as substrate and measuring the consumption of NADPH at 340 nm [36]. Briefly, aliquots of 250 μL of the homogenate were incubated in the presence of 5 μM FeSO_4_ and/or xanthone III and V (0.5, 1 and 2.5 μM). The final volume for all probes was 500 μL. All incubations were done at 37°C for 2 h in a shaking water bath. After incubation 50 μL of homogenate were mixed with 950 μL of the reaction mixture (1.25 mM GSSG, 0.1 mM NADPH, 0.5 mM Na_2_EDTA in 100 mM phosphate buffer, pH 7.6). One unit of GR was defined as the amount of enzyme that oxidizes 1 μmol NADPH/min. Data were obtained as units per milligram of protein and expressed as percent of GR activity vs. control.

### Statistical analysis

Results were expressed as mean values ± S.E.M. ROS scavenging capacity was expressed as 50% of the inhibitory concentration (IC_50_) value, which denotes the concentration of the xanthone and standard (μM) required to reach a 50% reduction in the respective molecule oxidation relative to the probe without xanthones or reference compound. IC_50_ was calculated by the least square method. The lower the IC_50_ value the higher the scavenging capacity of the compound. In regard to the experiments carried out in tissue homogenates, n = 6 experiments (each experiment corresponding to one rat) were considered. All data were analyzed by one-way ANOVA followed by Tukey’s test for multiple comparisons, using the Prism 3.02 software (GraphPad, San Diego, CA, USA). Values of P < 0.05 were considered as statistically significant.

## Results

### Scavenging activities of xanthones III and V

**Figure 2:**
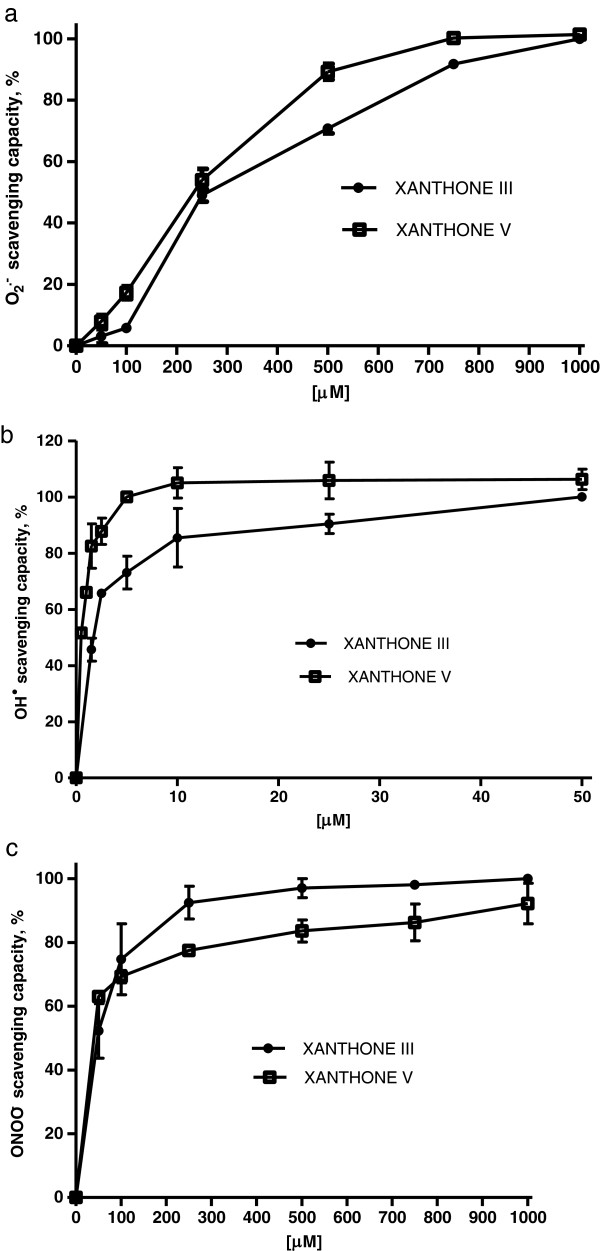
**Xanthones III and V are able to scavenge (a) O**_**2**_^**•−**^**, (b) OH**^**● **^**and (c) ONOO**^**− **^**in a concentration-dependent manner.***In vitro* systems of ROS production were assessed and subjected to different concentrations of xanthones (0.5-1,000 μM). Both xanthones showed an effective capacity as scavengers of O_2_^•−^, OH^●^ and ONOO^−^. Data are presented as mean values ± S.E.M. of seven experiments per group.

Xanthones III and V were able to scavenge O_2_^•−^ (Figure 2A), OH^●^ (Figure 2B) and ONOO^-^ (Figure 2C) in a concentration-dependent manner, in contrast, they were unable to scavenge H_2_O_2_ (data not shown). The IC_50_ values, calculated from the linear portion of the dose–response curve, are shown in Table 1. All these values are in the μM range. Xanthone V was more effective to scavenge ROS than xanthone III.

**Table 1:** ROS scavenging ability of xanthones III and V

	**O**_**2**_^**•—**^	**OH**^**●**^	**ONOO**^**—**^
Xanthone III	336.5 ± 8.9	1.82 ± 0.06	63.6 ± 11.6
Xanthone V	262.4 ± 7.7	0.07 ± 0.003	29.6 ± 6.8

Values are expressed as IC_50_ (μM).

Data are mean ± S.E.M. of six experiments per group. OH^●^: hydroxyl radicals, O_2_^●—^ : superoxide anion, ONOO^—^ : peroxynitrite anion.

### Xanthones III and V prevent the oxidative protein degradation in a concentration-dependent manner

**Figure 3:**
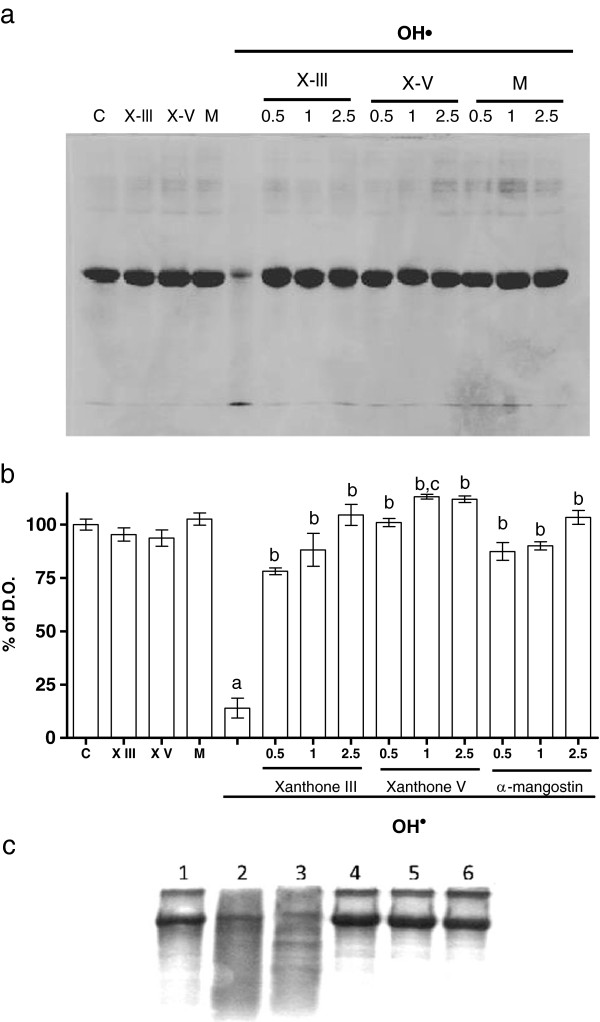
**Protective effect of xanthones III and V on bovine serum albumin (BSA) oxidation induced by an OH**^**● **^**generation system. a)** Representative Coomassie blue-stained-BSA gel is shown. Different concentrations (0.5, 1 and 2.5 μM) of xanthones III (X-III) and V (X-V) and α-mangostin (M) were tested in OH^●^ generator system. **b)** Quantitative representation of the BSA detection. **c)** Representative Coomassie blue-stained-BSA gel is shown. Lane 1, BSA; lane 2, OH^●^ + BSA; lane 3, EtOH (0.01%) + BSA + OH^●^; lanes 4, 5 and 6, xanthones III, V and α-mangostin (2.5 μM) respectively. + BSA + OH^●^. Data are presented as mean ± S.E.M. of 4 independent experiments. ^a^P < 0.01 vs. Control (C), ^b^P < 0.01 vs. BSA + OH^●^, ^c^P < 0.01 vs. α-mangostin.

**Figure 4:**
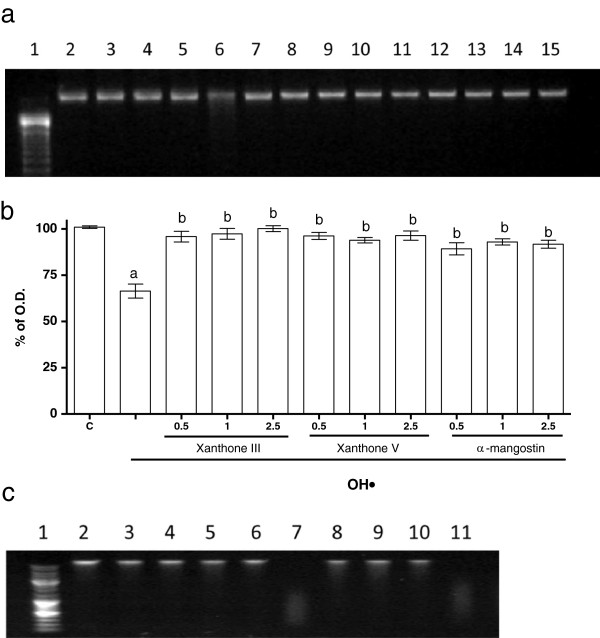
**Xanthones exerted a protective effect on DNA oxidation induced by an OH**^**● **^**generation system. a)** Representative ethidium bromide stained DNA-agarose gel. Lane 1, molecular weight markers; lane 2, DNA (20 μg); lane 3, xanthone III (2.5 μM); lane 4, xanthone V (2.5 μM); lane 5, α-mangostin (2.5 μM); lane 6, DNA (20 μg) + OH^●^; lanes 7–9 DNA (20 μg) + OH^●^ + xanthone III (0.5, 1 and 2.5 μM, respectively); lane 10–12 DNA (20 μg) + OH^●^ + xanthone V (0.5, 1 and 2.5 μM, respectively); lanes 13–15, DNA (20 μg) + OH^●^ + α-mangostin (0.5, 1 and 2.5 μM, respectively). **b)** Quantitative representation of the DNA detection. **c)** Representative ethidium bromide stained DNA-agarose gel. Lane 1, molecular weight markers; lane 2, DNA (20 μg); lane 3, xanthone III (2.5 μM); lane 4, xanthone V (2.5 μM); lane 5, α-mangostin (2.5 μM); lane 6, EtOH; lanes 7, DNA (20 μg) + OH^●^; lane 8, DNA (20 μg) + OH^●^ + xanthone III (2.5 μM); lane 9, DNA (20 μg) + OH^●^ + xanthone V (2.5 μM); lanes 10, DNA (20 μg) + OH^●^ + α-mangostin (2.5 μM) and lane 11, DNA (20 μg) + OH^●^ + EtOH. Data are presented as mean ± S.E.M of 4 independent experiments. ^a^P < 0.01 vs. Control, ^b^P < 0.01 vs. DNA + OH^●^.

Xanthones III and V (0.5, 1 and 2.5 μM; Figure 3) were able to prevent, in a concentration-dependent manner, the BSA degradation induced by OH^●^. A densitometric assessment was employed for protein detection. Protection was found since the lowest concentration of each xanthone used (0.5 μM), although this protection was more evident at higher concentrations (1 and 2.5 μM; Figures 3a and 3b). The EtOH did not have effect in the DNA degradation induced by OH^●^ (Figure 4c; line 3). These data confirm previous observations on the OH^●^-scavenging capacity of xanthones. α-Mangostin prevented BSA degradation induced by OH^●^ at all concentrations used.

### Xanthones III and V block DNA degradation induced by OH•

Figure 4a shows the protective effect of xanthones III (lanes 7–9) and V (lanes 10–12) on DNA degradation induced by OH^●^ (lane 6). The effect of α-mangostin was also evaluated in this paradigm (lanes 13–15), as a reference. The OH^●^ generator system induced around 35% of DNA degradation (Figure 4b), which was completely abolished by xanthones III and V and α-mangostin, an effect not dependent of the concentration used. Figure 4c shows that the three compounds tested did not exert any effect when compared to control group at the highest concentration used (2.5 μM; lanes 3, 4 and 5) and also shows that the EtOH at the maximum concentration used (0.01%) does not have any effect on the DNA degradation induced by OH^•^ (line 11).

### Xanthones III and V reduce the ROS production induced by FeSO_4_

**Figure 5:**
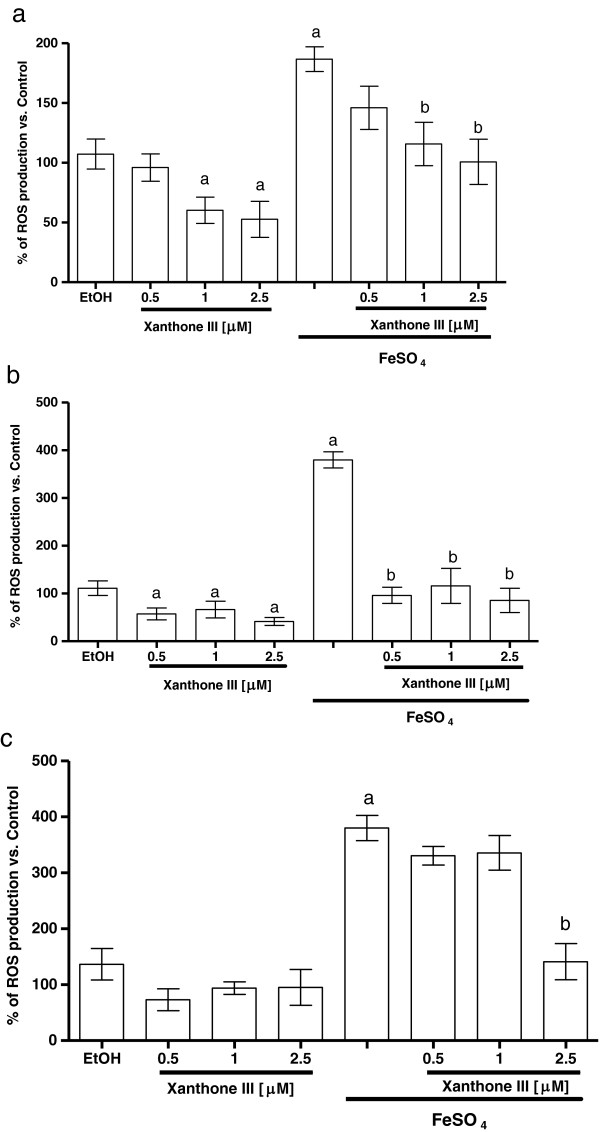
**Effect of xanthone III on the ROS production induced by FeSO**_**4 **_**in homogenates of different tissues. a)** Forebrain, **b)** liver and **c)** kidney homogenates were incubated with 5 μM FeSO_4_ and different concentrations on xanthone III (0 – 2.5 μM) in Kreb’s buffer. Control is represented by 100% and EtOH (50%) was used as a vehicle to dissolve xanthones. In all panels, mean values ± S.E.M. of six experiments per group are shown. ^a^P < 0.01 vs. control; ^b^P < 0.01 vs. FeSO_4_.

**Figure 6:**
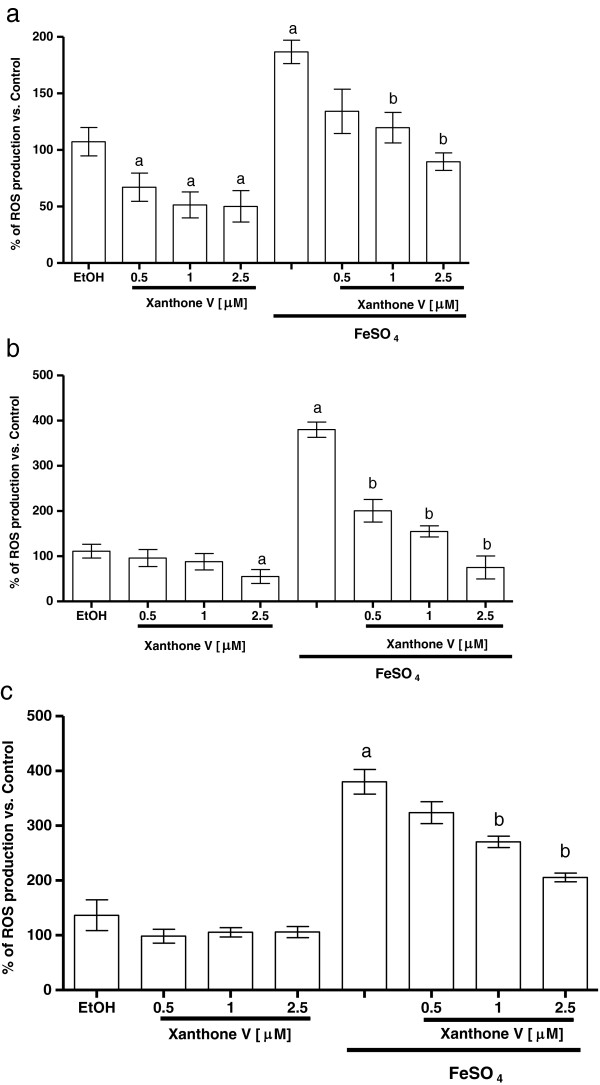
**Effect of xanthone V on the ROS production induced by FeSO**_**4 **_**in homogenates of different tissues. a)** Forebrain, **b)** liver and **c)** kidney homogenates were incubated with 5 μM FeSO_4_ and different concentrations on xanthone V (0 – 2.5 μM) in Kreb’s buffer. Control is represented by 100%. In all panels, mean values ± S.E.M. of six experiments per group are shown. ^a^P < 0.01 vs. control; ^b^P < 0.01 vs. FeSO_4_.

FeSO_4_ induced an increase in ROS formation in forebrain, liver and kidney (186.7% 380%, and 380.3% when compared to control group, respectively (Figures 5 and 6a, 5 and 6b and 5 and 6c). In all preparations, xanthones III and V (0.5, 1 and 2.5 μM; Figures 5 and 6, respectively) reduced ROS formation to basal levels and also decreased ROS formation in a concentration-dependent manner in the homogenates exposed to the ferrous iron. Upon these conditions, ethanol (used as vehicle) did not have any effect when compared to control group. Both xanthones showed the same effectiveness to decrease this marker.

### Xanthones III and V reduced the lipid peroxidation produced by FeSO_4_

**Figure 7:**
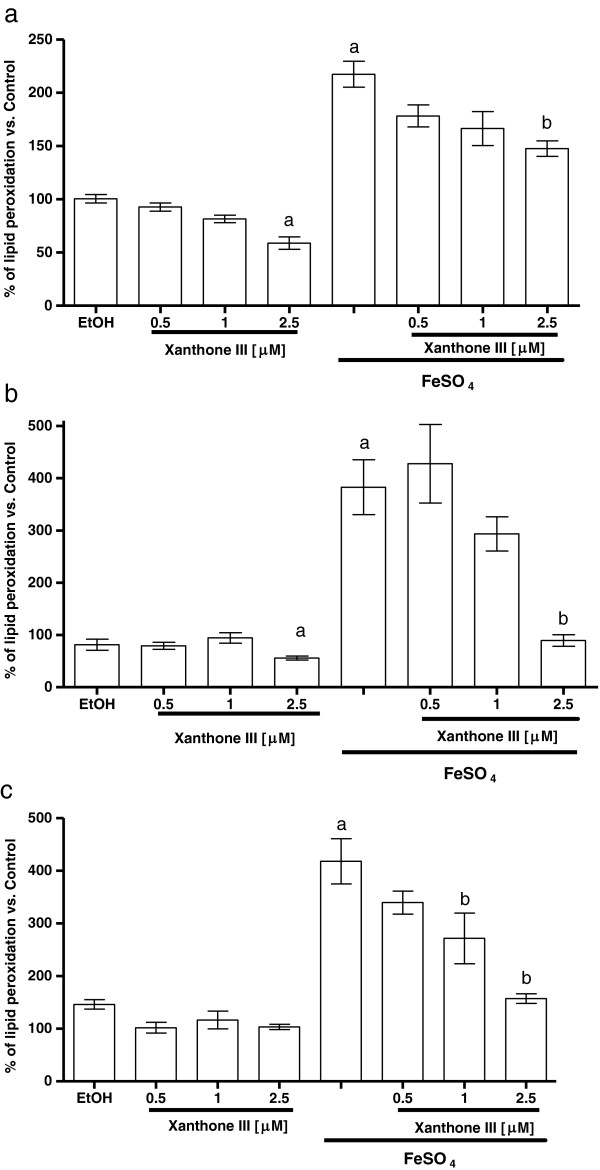
**Xanthone III decreased lipid peroxidation (LP) induce by FeSO**_**4**_**.** Different concentrations of xanthone III (0.5, 1, 2.5 μM) were incubated in the presence of ferrous iron in **a)** forebrain, **b)** liver and **c)** kidney homogenates. Control is represented by 100%. Data are presented as mean values ± S.E.M. of six experiments per group. ^a^P < 0.01 control; ^b^P < 0.01 vs. FeSO_4_.

**Figure 8:**
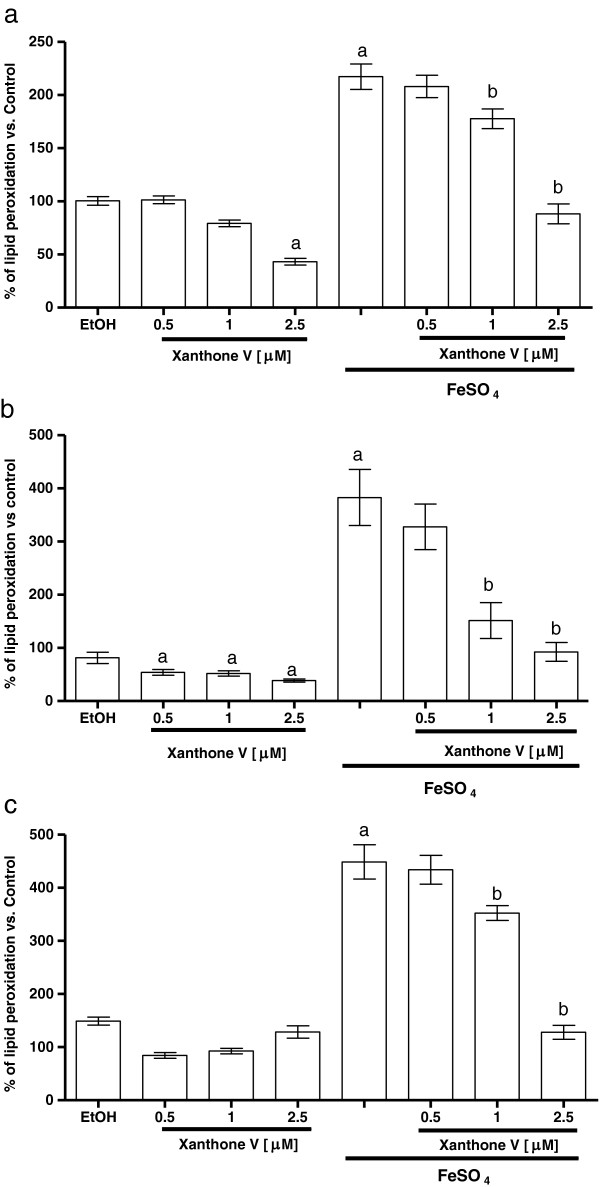
**Concentration-response effect of xanthone V on the lipid peroxidation (LP) induced by FeSO**_**4 **_**in homogenates. a)** Forebrain, **b)** liver and **c)** kidney homogenates were incubated with 5 μM FeSO_4_ and different concentrations of xanthone V (0, 0.5, 1 and 2.5 μM) in Kreb’s buffer. Control is represented by 100%. In all panels, mean values ± S.E.M. of six experiments per group are shown. ^a^P < 0.01 vs. control; ^b^P < 0.01 vs. FeSO_4_.

Figures 7 and 8 show the FeSO_4_-induced increase in LP in homogenates of (a) forebrain, (b) liver and (c) kidney (217.5%, 383% and 418.3% when compared to control group, respectively). Xanthones III and V decreased the FeSO_4_-induced LP in a concentration-dependent manner in all tissue homogenates tested (Figures 7 and 8, respectively). In addition, the incubation with vehicle alone did not exert any effect on this marker. However, both xanthones III and V reduced lipid peroxidation at the highest concentration tested (2.5 μM) when compared to control group in forebrain and liver homogenates.

### Xanthone III and V prevent the reduction of glutathione reductase activity induced by FeSO_4_

**Figure 9:**
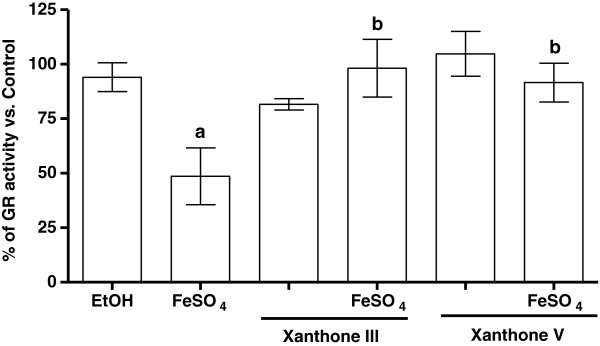
**Xanthones III and V prevented the glutathione reductase (GR) activity reduction induced by FeSO**_**4 **_**in brain homogenates.** Forebrain homogenates were incubated with 5 μM FeSO_4_ and 2.5 μM xanthones III or V in Kreb’s buffer. Control is represented by 100%. Mean values ± S.E.M. of three experiments per group are shown. The data are represented as a percentage with respect to control. ^a^P < 0.01 vs control; ^b^P < 0.01vs. FeSO_4_.

In brain homogenates, xanthones III and V prevented the decrease in GR activity generated by FeSO_4_ (~ 50% of reduction when compared to control group; Figure 9). In liver and kidney homogenates, ferrous iron did not have any effect in GR activity (data not shown). The vehicle alone showed no effects.

## Discussion

Several properties have been described for compounds derived from *Calophyllum brasiliense Cambess*[9,10,16]. Specially, xanthones exhibit diverse biological properties, including antihypertensive, antioxidative, antithrombotic and anticancer activities [37,38]. The xanthone nucleus is known as 9-xanthenone or dibenzo-γ-pyrone [39-41] and its biological activity depends on specific substituents and their positions to the basic structure in the ring system of each molecule [37]. The xanthones used here, III and V, are the most abundant extracted from the heartwood of *C. brasiliense*. In the present study, novel findings on ROS scavenging activity and protective effects were described for the antioxidant compounds xanthones III and V. Here, we found that both xanthones were able to scavenge O_2_^●—^, OH^●^ and ONOO^-^; under our assay conditions. It has been reported that glutathione show a higher IC_50_ for superoxide (2,171.5 μM) than the both xanthones employed in this work [42], and also we demonstrated that these xanthones are better OH^•^ –scavenger than α-mangostin (IC_50_: 8000 μM) [19,43]. Noteworthy, xanthone V showed a lower IC_50_ than xanthone III in all the synthetic scavenging assays. This effect could be due to the fact that xanthone V has an extra hydroxyl group in C-3 compared to xanthone III. Several authors have reported that the presence of hydroxyl groups is an important factor for their functionality [16,44,45]. The scavenging capacity of these xanthones is notorious since it has been widely reported that excessive amounts of ROS can affect, in a direct or indirect manner, some essential biomolecules such as DNA, proteins and lipids, further leading to cell death either through necrosis or apoptosis [46]. Also, this scavenging capacity supports the previous hypothesis postulated by Reyes-Chilpa [23], in which he proposed that xanthones III and V prevent the wood degradation by brown rot fungi that involves secretion of fungal H_2_O_2_ and its interactions with wood Fe^2+^ ions through their antioxidant and free radical scavenging properties.

We also showed that BSA and DNA degradation were prevented by both xanthones III and V, exhibiting the same efficacy than α-mangostin to prevent these changes. The degradation of BSA and DNA was promoted by the Fenton reaction, supporting the concept that plasma concentrations of transition metals such as copper or iron, can increase with age as well as in some pathologies such as diabetes. These metals readily catalyze reactions related to the formation of H_2_O_2_ through the Fenton reaction to form OH^●^, the most powerful oxidizing species able to interact directly with DNA and proteins.

Due to the scavenging capacity exerted by xanthones III and V, the potential protective effect of these molecules against FeSO_4_ was further studied in homogenates from rat brain, liver and kidney to elucidate if the protective effect depends of the nature of the tested tissue. Here we show that xanthones III and V can prevent an increase in the levels of the oxidative markers tested, which in turn are related with the scavenging capacity exhibited by these xanthones in synthetic assays.

Interestingly, the antioxidant effect showed by xanthones on ROS production and lipid peroxidation was not dependent on the tissue studied. Moreover, only in brain homogenates, FeSO_4_ was able to decrease the GR activity, and this effect was prevented by both xanthones. The fact that these xanthones can modulate the GR activity in the brain after the pro-oxidant insult was applied is relevant since the brain is known to be particularly vulnerable to oxidative damage due to its high rate of oxidative metabolic activity, high polyunsaturated fatty acid content, relatively low antioxidant capacity and inadequate neuronal cell repair activity [47]. In this context, GSH is considered the most important endogenous antioxidant in the brain, and GR plays an important role for the conversion of GSSG to GSH. Thus, the evidence collected here suggests that xanthones III and V can be considered as effective antioxidants since they can recover GR activity and were more efficient to scavenge O_2_^●—^ than GSH [42]. Thus, since it has been described that different xanthones can modulate a number of physiological molecules [6,14,16], our results suggest that the brain GR can also be a molecular target for xanthone III and V.

Considering that the tested xanthones can exert a protective action on some biomolecules from the oxidative damage in the same magnitude than α-mangostin, it is desirable to elucidate specific mechanisms and targets by which these molecules exert their protection, thereby raising expectations on its potential use for therapeutic purposes. Particularly, our group and others have made important descriptions on the properties of α-mangostin, a well-known xanthone derived from the pericarp of mangosteen fruit, which has antioxidant and anti-inflammatory properties [19,21,24,48-51]. This typically oriental product contrast with the endemic location of *Calophyllum brasiliense* Cambess, a species geographically located in Latin America. Therefore, the latter constitutes an alternative source of xanthones to be studied and used in alternative medicine around the Occidental hemisphere. Particularly, our study raises considerations on xanthones to be considered as an innovative tool with potential benefits for treatment of those diseases coursing with oxidative stress.

## Conclusions

In summary, this work provides evidence that the natural xanthones III and V possess potential antioxidant properties, although the antioxidant mechanisms through which they exert their actions need to be explored in detail in further studies.

## Abbreviations

ROS: Reactive oxygen species; O2●—: Superoxide; OH●: Hydroxyl radical; H2O2: Hydrogen peroxide; ONOO—: Peroxynitrite; xanthone III: Jacareubin; xanthone V: 2-(3,3-dimethylallyl)-1,3,5,6-tetrahydroxyxanthone; GR: Glutathione reductase; DCF: 2′,7′-dichlorofluorescein; DCFH-DA: 2′,7′-dichlorodihydrofluorescein diacetate; ABTS: 2,2′-azinobis-(3-ethylbenzothiazoline-6-sulfonic acid); DPPH: 2,2-diphenyl-1-picrylhydrazyl; BSA: Bovine serum albumin; DMSO: Dimethylsulfoxide; DTPA: DL-penicillamine, diethylenetriaminepentaacetic acid; NADPH: Nicotinamide adenine dinucleotide reduced form; LP: Lipid peroxidation; MDA: Malondialdehyde; GSSG: Oxidized glutathione; GSH: Glutathione.

## Competing interests

The authors declare that they have no competing interest. The authors alone are responsible for the content and writing of the paper.

## Authors’ contributions

BT, carried out the synthetic and biological assays. LR, carried out the DNA and Protein degradation. SEM and RR, carried out the xanthones extraction. RE, carried out some of the biological experiments. PB, analyzed and interpreted the data. MO and PE, carried out the enzymatic assays. SL and TC helped to perform the experiment. SD, performed the statistical analysis. PJ, contributed in drafting, revision the manuscript and obtained funding. RC, reviewed and commented on the manuscript for intellectual context. PV, conceived the idea for the study, wrote the body of the text and obtained funding. TM, conceived the idea and design of the study. All of the authors read the manuscript, contributed in correcting it and approving its final version.

## Pre-publication history

The pre-publication history for this paper can be accessed here:

http://www.biomedcentral.com/1472-6882/13/262/prepub
